# Sensitizing agents found in children and adolescents with recalcitrant atopic dermatitis: a cross-sectional study with a pediatric battery^☆^

**DOI:** 10.1016/j.abd.2021.08.005

**Published:** 2022-02-28

**Authors:** Cristiane Almeida Soares Cattani, Kenselyn Oppermann, Simone Perazzoli, Nathalia Hoffmann Guarda, Paula Baréa, Renan Rangel Bonamigo

**Affiliations:** a Sanitary Dermatology Outpatient Clinic, Secretaria da Saúde do Estado do Rio Grande do Sul, Porto Alegre, RS, Brazil; b Postgraduate Program in Pathology, Universidade Federal de Ciências da Saúde de Porto Alegre, Porto Alegre, RS, Brazil; c Faculty of Medicine, Universidade Federal do Rio Grande do Sul, Porto Alegre, RS, Brazil

**Keywords:** Adolescent, Child, Dermatitis, allergic contact, Dermatitis, atopic, Patch tests

## Abstract

**Background:**

Atopic dermatitis is the most common inflammatory skin disease in childhood and has an important impact on quality of life, especially severe cases or those that are recalcitrant to treatments. Sensitization to allergens with the potential for allergic contact dermatitis is a factor associated with cases of recalcitrant atopic dermatitis. Understanding the relationship between atopic dermatitis, allergens, and allergic contact dermatitis is essential. In Brazil, there are no studies on sensitization to allergens found in patch tests with pediatric batteries in patients with atopic dermatitis.

**Objectives:**

To verify the main sensitizing agents, the prevalence of allergic contact dermatitis and the epidemiological and clinical profile of children and adolescents with atopic dermatitis.

**Methods:**

Cross-sectional, prospective study in patients between 4 and 18 years of age, with recalcitrant atopic dermatitis, treated at the Sanitary Dermatology Outpatient Clinic (RS). All patients underwent patch tests with a battery of pediatric allergens.

**Results:**

The prevalence of sensitization and allergic contact dermatitis in the evaluated patients was 37.07% (20/54) and 27.7% (15/54), respectively. The most frequent allergens were: nickel sulfate (16.7%), disperse blue (5.6%), and fragrance mix I (5.6%). Nickel was associated with the female sex (p = 0.019).

**Study limitations:**

Sample size and selection, absence of a control group.

**Conclusions:**

A proportion of patients with recalcitrant atopic dermatitis may be sensitized to different allergens and may even have developed allergic contact dermatitis. Recognizing this context is important in the prevention strategy and management of the disease.

## Introduction

Atopic dermatitis (AD) and allergic contact dermatitis (ACD) are two of the most common inflammatory skin diseases in the pediatric population with a complex interrelationship.^1^, ^2^

The prevalence of AD has increased significantly over the past thirty years, affecting between 15% and 30% of children in Western countries.^3^ Likewise, ACD is frequent in both the adult and pediatric populations, including reports even in children of younger ages.^4^, ^5^ Recent studies have reported an increase in the prevalence of ACD in symptomatic children ranging from 27% to 95%, 6%.^5^, ^6^, ^7^, ^8^

Given the skin barrier dysfunction that decreases the protection against environmental agents, irritative contact dermatitis (ICD) and allergic contact dermatitis (ACD) are significant problems among individuals with AD.^5^, ^9^

Clinically, AD and ACD may be indistinguishable, and concomitant allergic reactions have been described as possible causes of recalcitrant or therapy-resistant atopic dermatitis.^1^, ^10^, ^11^

Although there are no evidence-based consensus guidelines for patch tests (PT) in children, it is a safe and useful tool for identifying skin sensitization.^5^, ^12^

Most studies on the prevalence and clinical and epidemiological characteristics of ACD in children and adolescents with AD are retrospective and come from North American and European centers.^1^, ^2^, ^5^

In Brazil, there are few studies on ACD in the pediatric population, and all of them performed PT using the standard Brazilian batteries used in adults or batteries of cosmetics; to date, there are no records of studies that have used PT pediatric batteries in Brazil.^7^, ^13^, ^14^, ^15^ It is worth emphasizing the importance of knowing the sensitizers in each country, given the variability of exposures and differences between regions of the world.

The present study aims to verify the sensitizers and the presence of ACD in children and adolescents with recalcitrant AD, in addition to their epidemiological and clinical profile, in a southern Brazilian sample that was submitted to a pediatric patch test battery series.

## Materials and methods

This is a prospective, cross-sectional study carried out with patch tests in patients with recalcitrant atopic dermatitis, aged between 4 and 18 years, treated at the Sanitary Dermatology Outpatient Clinic, Porto Alegre, State of Rio Grande do Sul (Brazil), between February 2019 and March 2020.

This study was approved by the Research Ethics Committees of the Health Secretariat of the State of Rio Grande do Sul and Universidade Federal de Ciências da Saúde de Porto Alegre (Counsels number 3,091,016 and 3,084,699, respectively). Only patients whose parents/legal guardians signed the consent form were evaluated. Anamnesis and physical examination were performed at the first consultation.

The selected patients were those diagnosed with atopic dermatitis, following the modified criteria of Hanifin and Rajka,^16^ and those who had a history of recalcitrant dermatitis (or dermatitis that recurred soon after discontinuation of topical treatment) and, therefore, had a clinical suspicion of allergic contact dermatitis.^10^, ^11^, ^12^ Patients using corticosteroids or topical immunomodulators within the last seven days, systemic immunosuppressants or corticosteroids within the last 30 days, those exposed to ultraviolet radiation 15 days before the test, those who had dermatitis at the test site, history of anaphylaxis, psychiatric disorders or pregnancy were excluded.^12^

The variables collected were: age, sex, age at onset and duration of dermatitis, presence of other associated atopic diseases, and family history of atopic diseases. Moreover, the following were evaluated: skin phototype, the topography of dermatitis, and AD severity through the Eczema Area and Severity Index (EASI) by body region (face and cervical region, upper limbs, lower limbs, and trunk), which can vary from 0% to 100%; Intensity of each lesion (erythema, edema or papules, excoriation and lichenification), on a scale of 0 to 3. The sum of the points classifies the disease as very mild (0.1 to 1); mild (1.1 to 7); moderate (7.1 to 20); severe (21.1 to 50); or very severe 50.1 to 72).^17^

PTs were performed using a modified pediatric battery, based on the consensus of the European Academy of Allergology and Clinical Immunology (EAACI) Task Force on Allergic Contact Dermatitis in Children on recommendations for patch testing with a battery of major allergens in the pediatric group.^12^ The battery was developed by the company IPI -ASAC Brasil^R^ and adapted according to the availability of the products in Brazil. It consists of 20 substances, including allergens and mixtures (Table 1).^18^ Some substances are present in the Brazilian standard battery,^19^ but others do not exist regionally, such as bufexamac (an anti-inflammatory agent) and Tixocortol pivalate (group A corticosteroid), which were removed. This was replaced by hydrocortisone (same group A). One component of the *Compositae* mixture was also excluded due to lack of the raw material. The allergens were inserted into 8-mm chambers in Alergo Chamber containers (Neoflex^R^).

**Table 1:** Brazilian pediatric battery – based on EAACI consensus.^12^, ^18^

Substance	Concentration. (%)	Vehicle
1- Hydrocortisone acetate	25.0	Vaseline
2- Lanolin	30.0	Vaseline
3- Disperse blue	1.0	Vaseline
4- Potassium bichromate	0.5	Vaseline
5- Budesonide	0.1	Vaseline
6- P-tert-butylphenol formaldehyde resin	1.0	Vaseline
7- Colophony	20.0	Vaseline
8- Compositae mix	1.9	Vaseline
9- Fragrance mix II	14.0	Vaseline
10- Mercaptobenzothiazole	2.0	Vaseline
11- Lyral	5.0	Vaseline
12- Mercapto mix	2.0	Vaseline
13- Methylchloroisothiazolinone/Methylisothiazolinone	1.0	Water
14- Methyldibromo glutaronitrile	1.0	Vaseline
15- Neomycin	20.0	Vaseline
16- P-phenylenediamine	0.5	Vaseline
17- Fragrance mix I	7.0	Vaseline
18- Sesquiterpene Lactone Mix	0.1	Vaseline
19- Nickel sulfate	5.0	Vaseline
20- Tiuram mix	1.0	Vaseline

The procedures were performed according to the recommendations of the International Contact Dermatitis Group (ICDRG).^12^ The containers were applied to the dorsal area of the patients and removed within 48 hours. The readings were performed at 48 hours and at 96 hours, both by the same dermatologists. Tests with reactions classified as +, ++ or +++ were considered positive. Only the results of the 96 -h readings were included in the statistical analysis. Patients with ACD were considered to be those who tested positive within 96 hours with currently relevant allergens (exposure to allergens that explained the clinical picture at the time). Those with no current relevance were classified as sensitized but not having ACD.

Data analysis was performed using the SPSS software, version 25. The chi-square test or Fisher’s test were used for categorical variables. For continuous variables, the Mann-Whitney test was used. Values ​​of p < 0.05 were considered statistically significant.

## Results

Fifty-four patients with AD were evaluated. Using the World Health Organization classification,^7^ the sample was divided into two age groups: children (4 to 9 years old) and adolescents (10 to 18 years old), of which 31 (57.4%) were children and 23 (42.6%) were adolescents (Table 2). The female sex predominated with 35 patients (64.8%), as well as phototypes III and IV, with 15 (27.8%) and 21 (38.9%) patients, respectively. The mean age of AD onset was 2.63 years (SD = 2.7), with a median of 1.5 years. The mean duration of AD was 6.57 years (SD = 4.2) with a median of 5 years.

**Table 2:** Atopic dermatitis and patch tests: sample characteristics (n = 54) and associations.

Variables	Category(n)	Negative tests (n)	Positive tests n (%)	Statistical test	p-value
Sex	Male (19)	17	2 (10.5)		
	Female (35)	22	13 (37.1)	χ^2^=3.123	0.077
Age group	4–9 y (31)	20	11 (35.5)		
	10–18 y (23)	19	4 (17.5)	χ^2^=1.347	0.246
Phototype	I (2)	1	1 (50)		
	II (4)	4	0		
	III (15)	11	4 (26.6)		
	IV (21)	14	7 (33.3)	χ^2^=5.46	0.361
	V (11)	9	2 (18.2)		
	VI (1)	0	1 (100)		
Personal atopy^a^	Yes (45)	32	13 (28.8)	Fisher	1.0
	No (9)	7	2 (22.2)		
Familial atopy	Yes (35)	23	12 (34.2)	χ^2^=1.279	0.258
	No (19)	16	3 (15.8)		
EASI score	Mild (19)	14	5 (26.3)		
	Moderate (25)	18	7 (28)	χ^2^= 0.544	0.901
	Severe (9)	6	3 (33)		
	Very severe (1)	1	0		
Site of dermatitis	Face/Cervical area (34)	26	8 (23.5)	χ^2^= 0.826	0.363
	UL (46)	35	11 (23.9)	Fisher	0.197
	Trunk (37)	25	12 (32.4)	Fisher	0.338
	LL (52)	39	13 (25)	Fisher	0.073
Duration of AD (years)	Mean	7,3	4.5	MW-U= 154	0.007
	SD	4,2	3.6		
	Median	6	3.2		
Age at AD onset (years)	Mean	2,7	2.3	MW-U =274	0.720
	SD	2,8	2.5		
	Median	2	1		

y, years; UL, Upper Limbs; LL, Lower Limbs; SD, Standard Deviation; χ^2^,Chi-square; MW-U, Mann-Whitney.

a Other atopic diseases (in addition to AD).

Other atopic diseases were present in 45 patients (83.3%), and there was a family history of atopy in 35 patients (64.8%).

Regarding AD severity, most patients had mild and moderate EASI (35.2% and 46.3%, respectively). Nine (16.7%) had severe disease, and there was only one with very severe AD. The most affected body areas were: lower limbs (96.3%), upper limbs (85.2%), trunk (68.5%), face and cervical area (63%). Dermatitis affected three or more areas in 42 patients (78%).

Twenty 96 -h positive reactions were recorded in the 54 tested patients, that is a sensitization rate of 37.07% was found (20/54). The relevance index was 75%; that is, in 15 patients the current exposure to the sensitizer accounted for the current clinical picture. Therefore, the prevalence of ACD in the present sample was 27.7% (15/54; Table 2, Table 3).

**Table 3:** Allergen profile: positive tests in relation to sex and age groups.

Allergens	PPT	M	F	p-value	4–9 y	10–18 y	p-value	Clinical relevance
	n = 20 (%)	n = 19 (%)	n = 35 (%)		n = 31 (%)	n = 23 (%)		
Nickel sulfate	9 (16.7)	0	9 (25.7)	0.019	7 (22.6)	2 (8.7)	0.273	Yes
Disperse blue	3 (5.6)	2 (10.5)	1 (2.8)	0.280	3 (9.7)	0	0.253	Yes
FM I	3 (5.6)	0	3 (8.6)	0.544	2 (6.4)	1 (4.3)	1.000	Yes
Neomycin	2 (3.7)	0	2 (5.7)	0.535	2 (6.4)	0	0.502	Yes
Formaldehyde	1 (1.9)	0	1 (2.8)	1.000	1 (3.2)	0	1.000	No
FM II	1 (1.9)	0	1 (2.8)	1.000	0	1 (4.3)	0.426	Yes
MCI/MI	1 (1.9)	0	1 (2.8)	1.000	1 (3.2)	0	1.000	No

PPT, Number of Positive Patch Tests; M, Male; F, Female; y, years; FMI, Fragrance Mix I; FM II; Fragrance mix II; MCI/MI, Methylchloroisothiazolinone/Methylisothiazolinone.

OBS: Other battery allergens, all of them with negative results: Tiuram mix, Colophony, Mercaptobenzothiazole, Sesquiterpene Lactone Mix, Lyral, P-tert-butylphenol formaldehyde resin, Potassium bichromate, Lanoline alcohol, - P-phenylenediamine, Composite mix, Hydrocortisone acetate, Budesonide, Methyldibromo glutaronitrile.

It was verified that 11 of the 31 children (35.5%) and 4 of the 23 adolescents (17.4%) had positive tests, but this difference did not reach statistical significance (p = 0.107). Similarly, the statistical tests did not show greater sensitization associated with the following variables: phototype, personal or family history of atopy, age at AD onset, location, and severity of the disease. The female sex showed a statistical trend towards more positive tests (p = 0.077). A shorter duration of AD (mean = 4.5 years; SD = 3.60 [median 3.2 years]) was associated with a higher frequency of positive tests when compared with a longer duration of AD (mean = 7.3 years, SD = 4.25 [median 6 years]), with p = 0.007 (Table 2).

The allergens with the highest frequency of sensitization were: nickel sulfate (16.7%), disperse blue (5.6%), and fragrance mix I (5.6%). The overall profile of the allergens is shown in the table (Table 3). No allergens were associated with a specific age group; however, an association was demonstrated between nickel sensitization and the female sex (p = 0.19; Table 3).

The “angry back syndrome” was detected in one child. Irritant reactions appeared in 11 patients (20.4%), 10 of which were children (between 4 and 9 years old). Fragrances and nickel were the most common irritating substances among children. There were no adolescents with occupational exposure and only one, aged 16 years, showed an irritative reaction (to fragrance mix II).

## Discussion

The relationship between AD and ACD is yet to be fully understood.^1^, ^5^, ^20^

The Pediatric Contact Dermatitis Registry (PCDR), which contains data from the largest number of pediatric PTs in the US, reported that among 49% of children referred with AD, 42% also had ACD.^2^

Overall, most authors have not found significant differences in the prevalence of ACD between atopic and non-atopic children and adolescents.^2^, ^4^, ^8^, ^13^^,^^15^, ^21^, ^22^, ^23^ However, many of the published studies have shown controversial results regarding the subject. Sensitization rates varied considerably, as well as the study design in relation to sample size and characteristics, diagnostic criteria for AD, PT methodology, and allergens used. Additionally, a selection bias may have occurred in most studies because children without AD were more often referred for testing due to suspected ACD, whereas those with AD may have been referred because of AD recurrence.^1^ Few authors have published the PT results and the profile of children and adolescents with AD.^2^, ^3^, ^6^, ^14^^,^^24^, ^25^, ^26^, ^27^, ^28^, ^29^, ^30^, ^31^, ^32^, ^33^, ^34^, ^35^

Regardless of the fact that children with AD have an increased risk of sensitization in comparison to others, ACD can coexist in up to 30% of children with AD, according to recent publications.^1^, ^2^, ^11^

The batteries used in pediatric PT studies show geographic variations in allergen selection, but most are from North America and Europe.^1^, ^5^, ^7^ This is the first Brazilian study to assess the prevalence and profile of ACD in children and adolescents with AD through PT with a pediatric battery. In this sample, the prevalence of ACD was 27.7%, coinciding with the latest reviews of the international literature, in which the prevalence ranged from 20% to 44% in this group.^1^, ^5^, ^7^ Other Brazilian authors found a similar prevalence to the present one, in non-extensive, regionalized samples, but using the tests of the Brazilian standard battery (30 substances), not with a pediatric battery.^13^, ^14^, ^15^

Some studies have found a high rate of tests with irritant (false-positive) reactions in AD patients, probably due to a lower irritability threshold in a defective skin barrier.^5^, ^9^, ^14^, ^36^ In the present study, irritative reactions also appeared, in 20.4% of children, mainly to nickel and fragrances, in the younger age group. However, although false-positive reactions are frequent in AD patients, weak positive reactions may occur and should be considered true positive ones, especially in patients with AD exacerbation.^20^ Moreover, many patients also had relevant weak positive reactions, as in a Brazilian pediatric study that reported 84.2% of relevant positive PTs with 1+ intensity reactions.^15^

In the present study, 57.4% of the sample were children between 4 and 9 years of age, and although it is not significant, more positive results were found in this younger age group. This finding is in accordance with other authors reports of high sensitization rates in children from younger age groups.^2^, ^4^, ^31^ On the other hand, as allergen exposure increases with age, more cases of ACD were reported in the group of adolescents in several studies.^7^, ^13^, ^15^ Additionally, in relation to age, significant differences are described with some allergens.^7^ A large US study reported that younger children were more sensitized to ingredients in personal hygiene products, while sensitization to disperse blue and gold was more common in older children.^26^

In the present sample, the age of AD onset in children with positive PTs did not differ statistically from those with negative PTs, in line with the findings of Labadie et al.^31^ Likewise, the present study found no association between phototype and more positive PTs (p = 0.361). Few studies have included this variable, and one has suggested that ethnic and cultural trends, such as body piercing, could influence the incidence of ACD.^26^

Similar to several studies, the present investigation also observed a higher number of referred female patients (64.8%) and a greater tendency towards positive PTs (p = 0.077).^2^, ^13^, ^15^, ^21^^,^^26^

In the present study, a shorter duration of AD (median 3.2 years) was associated with more positive tests than a longer duration. This may be due to the predominance of referred younger children (57.4% of those aged between 4 and 9 years). The largest North American pediatric study also found more positive PTs in those with dermatitis of shorter duration.^26^

The association between the presence of other atopic diseases or family history and greater sensitization was not observed in the present sample. Other studies did not report any significant differences between atopy and age groups with positive PTs.^7^, ^8^, ^25^

Most of the patients in the present study had AD lesions in more than two body regions, as in the study by Rodrigues et al.^15^ Other authors have also reported more diffuse (spread) dermatitis among the tested children.^2^, ^7^

Significant associations have been described between AD, ACD, and hand eczema in adolescents.^1^, ^9^, ^35^

In the present study, as there were more patients with mild to moderate AD, there was no possible correlation between higher disease severity and greater allergic sensitization. Similarly, Ozceker et al. did not find more ACD in children with severe AD.^27^ However, other authors have reported an association between more positive PTs and more severe disease.^3^, ^32^, ^34^

In fact, some studies have suggested that the sensitization threshold would be inversely related to AD severity. A lower positivity in PT reactions in patients with severe AD could be explained by the increase in T helper 2 (Th2) cells, as opposed to the deviation of Th1 cells from the immune system, traditionally observed in ACD.^9^, ^10^, ^30^

### Allergen sensitization profile

The results of studies on the prevalence of ACD and the frequency of sensitizing allergens in children and adolescents with AD showed variations according to the selected sample, exposure time, type, and amount of tested substances.^5^, ^7^, ^37^

It is important to emphasize that there are differences in the allergen selection of the PT batteries used in pediatric studies worldwide;^7^ however, the most frequently applied are those of the North American Contact Dermatitis Group (NACDG)^21^ and the Pediatric Contact Dermatitis Registry (PCDR).^26^ Many studies in Europe have used standard or regional batteries according to the European Surveillance System on Contact Allergies (ESSCA).^38^, ^39^

Recently, based on a consensus survey, the American Contact Dermatitis Society proposed a pediatric battery for children aged 6 to 18 years.^39^, ^40^

However, these proposed batteries are not specifically targeted at children with AD. The allergen sensitization profile seems to be different in this population.^3^, ^5^, ^11^, ^23^

In the present study, all AD patients were tested with a pediatric battery, following the recommendations of the EAACI,^12^ but adapted to the Brazilian reality (substitution of three substances). The allergens with the highest sensitization rates, in order of frequency, were: nickel sulfate, disperse blue, fragrance mix I, and neomycin.

A recent US publication on ACD in children highlighted the main allergens in those with AD: nickel, fragrance mix I, Balsam of Peru, bacitracin, lanolin, formaldehyde, and neomycin.^2^

Teo et al. (2019), in an extensive review of studies comparing ACD in individuals without AD and with AD, highlighted the ten most common allergens in the latter: nickel, fragrance mix I, methylisothiazolinone (MI), cobalt, colophony, neomycin, p-phenylenediamine (PPD) thiuram mix, balsam of Peru, and sodium metabisulfite.^37^ Three of these allergens (nickel, fragrances and neomycin) were also the most frequently found in the present study.

Patients with AD may be at increased risk of sensitization due to a defective barrier and frequent use of topical products (skincare and medications).^2^, ^9^, ^2^, ^37^ Additionally, a large multicenter study (comprising 11 European countries) suggested that children with recalcitrant AD should also be tested for metals, disperse dyes, and *Compositae*.^38^

As found in several publications, in the present study, nickel was also the most sensitizing allergen^5^, ^14^, ^26^, ^28^^,^^29^, ^31^ and the most frequently associated with the female sex (p = 0.019).^8^, ^11^, ^13^, ^31^^,^^33^ Nickel sensitization has been related to the habit of ear piercing. Toys, clothing (zippers, buttons, etc.), electronic equipment (tablets and cell phones) are continuous sources of nickel release.^8^, ^11^

Disperse blue dye was the second most frequent allergen in the present study. Disperse dyes, present in brightly colored clothes and in some children's cosmetics, are potential allergens in children.^6^, ^12^ Although such substances are not part of the Brazilian standard battery, their importance in children with AD has been mentioned in recent studies.^22^, ^25^, ^26^

Emollients are the main skincare products used in the treatment of AD. Contact dermatitis to fragrances and preservatives contained in these emollients may play a role in aggravating the disease.^1^, ^3^, ^11^, ^12^ Fragrance mix I and II were found, in order of frequency, as the third and fifth allergens, respectively, in the present study. Fragrances are among the main substances present in toys and other scented products aimed at children, even in those labeled “fragrance-free”, which may contain related ingredients.^26^

Neomycin, the fourth most frequent allergen in the present study, is an antibiotic that is widely present in topical preparations and medications and has long been among the top allergens in children.^11^, ^21^, ^25^

Individuals with AD may be more sensitized to preservatives.^37^ Sensitization to methylchloroisothiazolinone, methylisothiazolinone, and the MCI/MI association present in cosmetics, cleaning and hygiene products has been increasing in recent studies.^8^, ^28^ Formaldehyde and formaldehyde releasers are also common preservatives in everyday products^11^ and are among the top five allergens in a large pediatric AD study.^2^ However, in the present study, only one patient showed sensitization to formaldehyde and one to the MCI/MI association.

Although several authors have mentioned allergy to topical corticosteroids, mainly in patients with treatment-resistant AD, the present sample did not show positive tests to less potent corticosteroids (group A) present in the pediatric battery. Some studies attribute this negative result to the fact that patients with the recalcitrant disease more frequently use the higher potency corticosteroids (groups B and D).^10^

Considering that certain allergens seem to show specific patterns according to age,^7^, ^21^, ^26^ some authors suggest the use of PTs with different batteries aimed at children and adolescents:^28^ reduced batteries in children (due to smaller body surface area)^4^, ^6^, ^25^, ^29^ and extended batteries in adolescents (greater exposure, including occupational).^28^, ^40^

However, the authors agree on the difficulty of designing a battery of ideal substances to be used in the screening of the pediatric population and recommend the use of regional and substance-customized batteries from products used by the patients based on exposure history and clinical relevance.^5^, ^28^, ^39^, ^40^

The strength of this study is that it is the first one in Brazil (to the best of our knowledge) to perform patch tests in children with AD using a pediatric battery to assess the prevalence, sensitizer, and ACD profile in this vulnerable group. The main limitations of this research could be the sample size, lack of a control group to be tested with the Brazilian standard battery to compare with other studies, and the fact that these patients came from a public health service (socioeconomic influences on exposure to certain allergens ). As this is a study that opens up the discussion on the use of pediatric batteries in recalcitrant atopic dermatitis, the cross-sectional design is the design that corresponds to the objectives.

## Conclusions

ACD can coexist and play a role in the worsening of AD. Almost one-third of children and adolescents with AD in a southern Brazilian sample attended in the public health system were diagnosed with ACD, using a pediatric patch test battery. The most important allergens in this population were nickel sulfate, disperse blue, fragrances, and neomycin. Future multicenter studies with larger samples, control groups, and standardized methodologies are required.

The present study adds information about sensitizers in children and adolescents with recalcitrant AD.

## Financial support

None declared.

## Authors’ contributions

Cristiane Almeida Soares Cattani: Statistical analysis; approval of the final version of the manuscript; design and planning of the study; drafting and editing of the manuscript; collection, analysis, and interpretation of data; effective participation in research orientation; intellectual participation in the propaedeutic and/or therapeutic conduct of the studied cases; critical review of the literature; critical review of the manuscript.

Renan Rangel Bonamigo: Statistical analysis; approval of the final version of the manuscript; design and planning of the study; drafting and editing of the manuscript; collection, analysis, and interpretation of data; effective participation in research orientation; intellectual participation in the propaedeutic and/or therapeutic conduct of the studied cases; critical review of the literature; critical review of the manuscript.

Kenselyn Oppermann: Approval of the final version of the manuscript; collection, analysis, and interpretation of data; effective participation in research orientation; intellectual participation in the propaedeutic and/or therapeutic conduct of the studied cases; critical review of the literature.

Simone Perazzoli: Approval of the final version of the manuscript; collection, analysis, and interpretation of data; intellectual participation in the propaedeutic and/or therapeutic conduct of the studied cases; critical review of the literature.

Nathalia Hoffmann Guarda: Approval of the final version of the manuscript; collection, analysis, and interpretation of data; intellectual participation in the propaedeutic and/or therapeutic conduct of the studied cases; critical review of the literature.

Paula Baréa: Approval of the final version of the manuscript; collection, analysis, and interpretation of data; intellectual participation in the propaedeutic and/or therapeutic conduct of the studied cases; critical review of the literature.

## Conflicts of interest

None declared.
